# 4D Arctic: A Glimpse into the Structure and Evolution of the Arctic in the Light of New Geophysical Maps, Plate Tectonics and Tomographic Models

**DOI:** 10.1007/s10712-013-9254-y

**Published:** 2013-10-18

**Authors:** Carmen Gaina, Sergei Medvedev, Trond H. Torsvik, Ivan Koulakov, Stephanie C. Werner

**Affiliations:** 1 Centre for Earth Evolution and Dynamics (CEED), University of Oslo, Oslo, Norway; 2 Geodynamics, Geological Survey of Norway, Trondheim, Norway; 3 School of Geosciences, University of Witwatersrand, WITS, Johannesburg, 2050 South Africa; 4 Trofimuk Institute of Petroleum Geology and Geophysics, SB RAS, Novosibirsk, Russia

**Keywords:** Arctic region, Magnetics, Gravity, Tomography, Mantle plume, Volcanism

## Abstract

Knowledge about the Arctic tectonic structure has changed in the last decade as a large number of new datasets have been collected and systematized. Here, we review the most updated, publicly available Circum-Arctic digital compilations of magnetic and gravity data together with new models of the Arctic’s crust. Available tomographic models have also been scrutinized and evaluated for their potential to reveal the deeper structure of the Arctic region. Although the age and opening mechanisms of the Amerasia Basin are still difficult to establish in detail, interpreted subducted slabs that reside in the High Arctic’s lower mantle point to one or two episodes of subduction that consumed crust of possibly Late Cretaceous–Jurassic age. The origin of major igneous activity during the Cretaceous in the central Arctic (the Alpha–Mendeleev Ridge) and in the proximity of rifted margins (the so-called High Arctic Large Igneous Province—HALIP) is still debated. Models of global plate circuits and the connection with the deep mantle are used here to re-evaluate a possible link between Arctic volcanism and mantle plumes.

## Introduction

Prompted by recent climate change and economic motives, the interest for studying the Arctic has exponentially increased in the last decade. A wealth of new geophysical and geological data has been collected, older classified data have been made publically available, and several international efforts have contributed to large, regional data set compilations. This data collection indicates a much more complicated structure of the Arctic crust than previously thought (e.g., Mosher et al. 2012). As our knowledge about detailed structures of this remote region is increasing, new concepts and integrated data sets have to be employed for modelling its tectonic evolution. In particular, mantle-lithosphere connections may hold some of the keys to deciphering the timing and mechanisms of opening and closing oceanic basins for the last 200 million years.

**Fig. 1:**
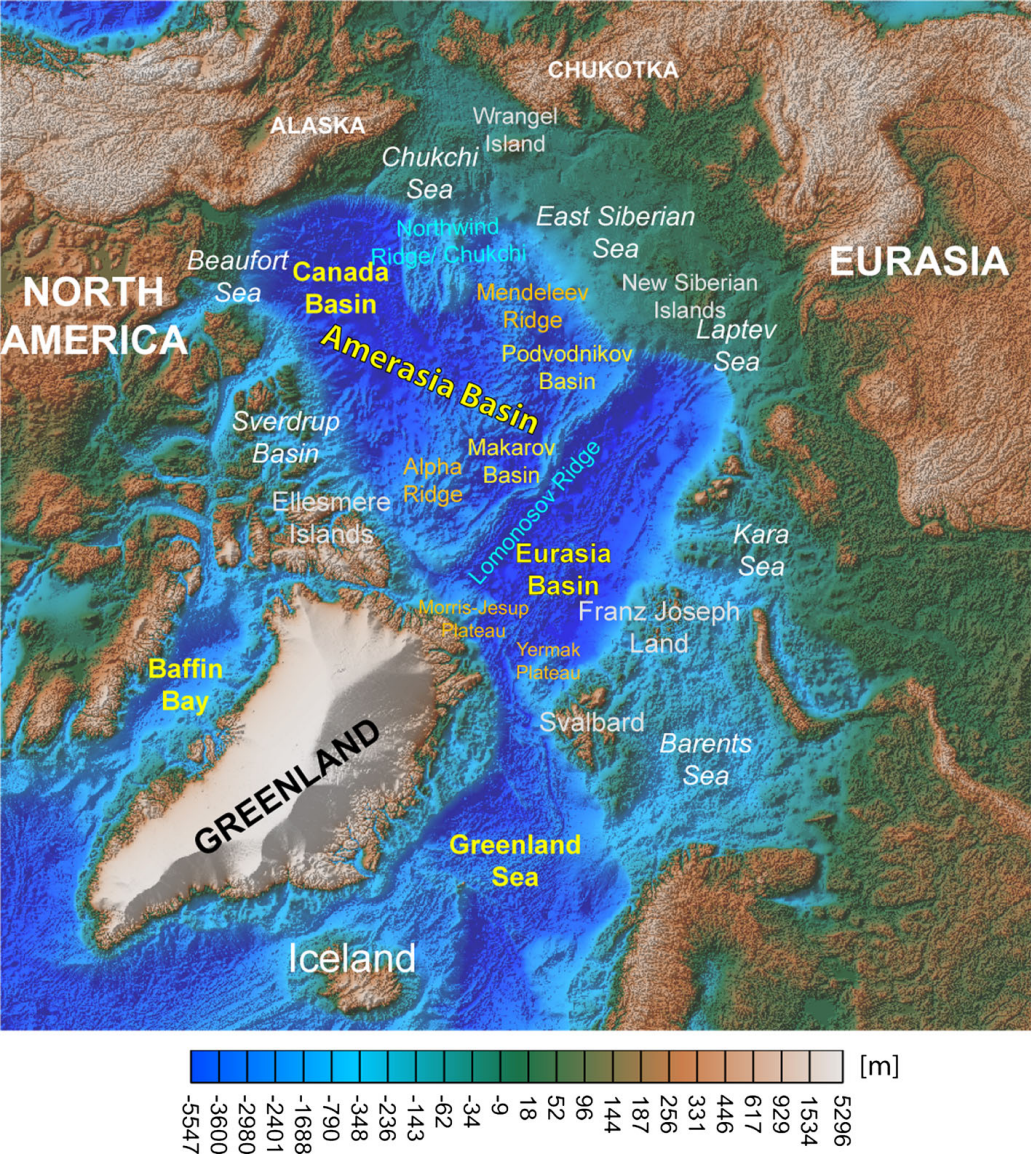
Topography and bathymetry (ETOPO1, Amante and Eakins 2009) of the Circum-Arctic region and nomenclature of main tectonic units

The present day Circum-Arctic region comprises a variety of tectonic settings: from active seafloor spreading in the North Atlantic and Eurasian Basin, and subduction in the North Pacific, to long-lived stable continental platforms in North America and Asia (Fig. 1). A series of rifted margins, abandoned rifted areas and presumably extinct oceanic basins fringe these regions. Moreover, rifting- and seafloor spreading-related processes formed many continental splinters and terranes that were transported and docked at higher latitudes. Volcanic provinces of different ages have also been identified, from the Permian–Triassic Siberian traps at ~251 Ma to the (presumably) Cretaceous HALIP and smaller Cenozoic provinces in northern Greenland and the Barents Sea.

Despite several decades of research, a consensus towards a model to explain all the geological and geophysical observations has not yet been reached. There are at least half a dozen kinematic models proposed for the evolution of the Amerasia Basin (Fig. 1)—an enigmatic submerged area that occupies more than half of the High Arctic region. Even the more recent evolution of the Arctic is not satisfactorily unravelled, and although a Mid–Late Paleocene age for the oceanic Eurasia Basin has been postulated, the exact timing of break-up is not known, nor are the plate boundary configurations mainly because the Eocene collision in SW Ellesmere/North Greenland has modified these plate boundaries.

In this contribution, we aim to demonstrate how recent data sets and models may change our view on the tectonic evolution of the Arctic. In Sect. 1, we will succinctly present the new geophysical maps that are used for the interpretation of main tectonic features in the Arctic region. A model for the evolution of the oceanic crust in the Eurasia and Amerasia Basins is developed in Sect. 2, together with a review of Iceland plume path models and possible connections with volcanism in the High Arctic. Section 3 reviews several tomographic models and speculates on the origin of observed mantle heterogeneities.

## New Regional Maps of the Arctic

**Fig. 2:**
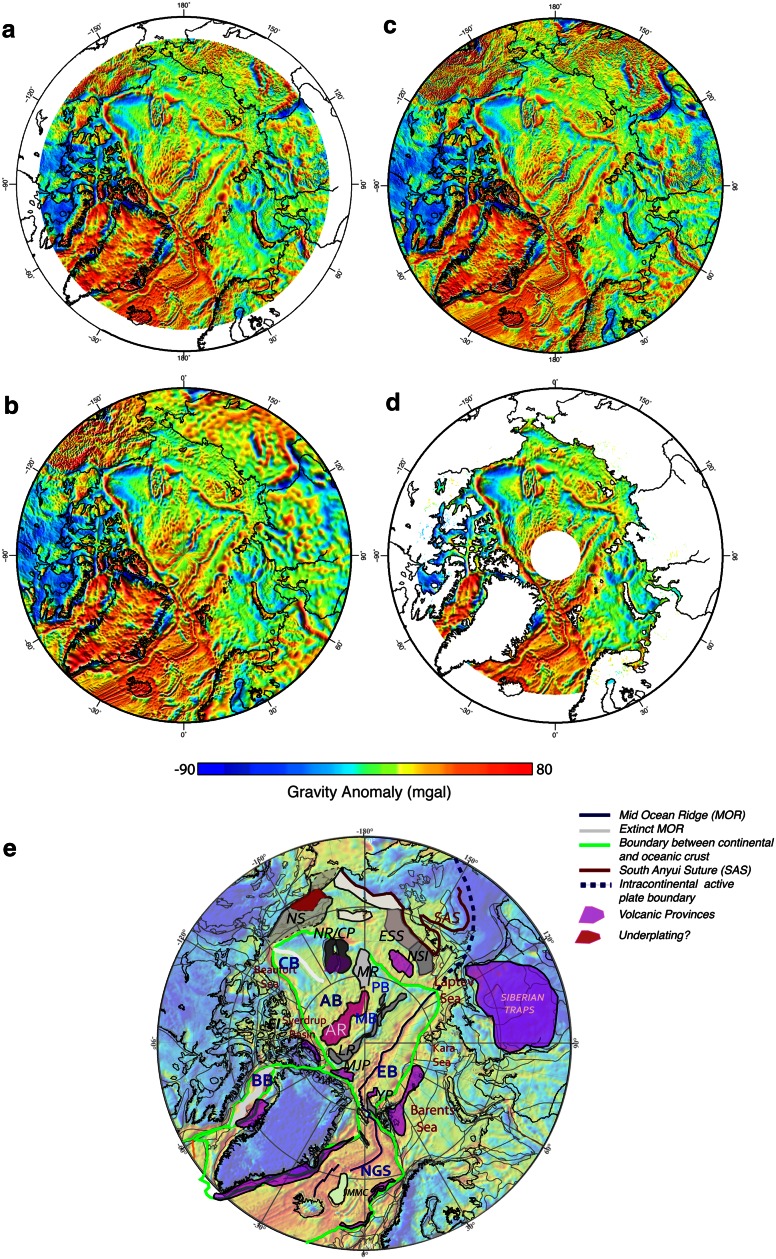
Recent gravity anomaly maps of the Arctic: **a** ArcGP, **b** CAMPGM-G, **c** DTU10 and **d** ARCS-2 (see Table 1 for details) **e** Interpretation of main tectonic features [the CAMPGM gravity map, (Gaina et al. 2011) is shown in the background]. Microcontinents: *JMMC* Jan Mayen, *LR* Lomonosov Ridge, *MS* Marvin Spur, *MR* Mendeleev Ridge, *NR*/*CP* Northwind Ridge/Chucki Plateau, *NSI* North Siberian Islands, *WI* Wrangel Islands, *NS* North Slope, *CT* Chukotka Terrane, *ESS* East Siberian Shelf; Oceanic Basins: *NGS* Norwegian Greenland Sea, *EB* Eurasia Basin, *MB* Makarov Basin, *PB* Podvodnikov Basin, *AB* Ameriasia Basin, *CB* Canada Basin

**Fig. 3:**
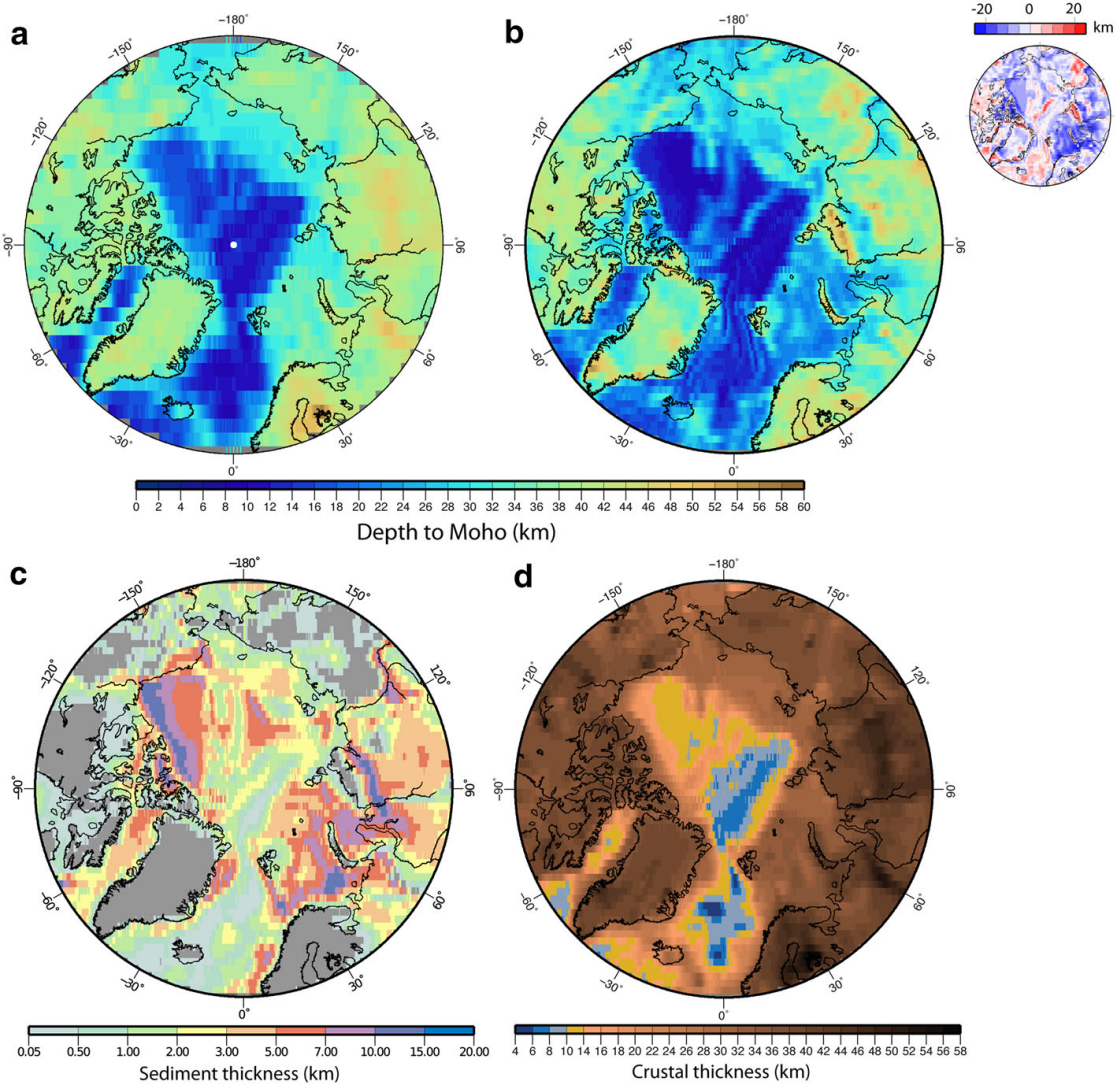
**a** Moho depth from CRUST1.0 model (Laske et al. 2013) and **b** from GOCE satellite data (Reguzzoni and Sampietro 2012); the difference between the two models is shown in the *right upper* corner, **c** sediment thickness and **d** crustal thickness (CRUST1.0)

**Fig. 4:**
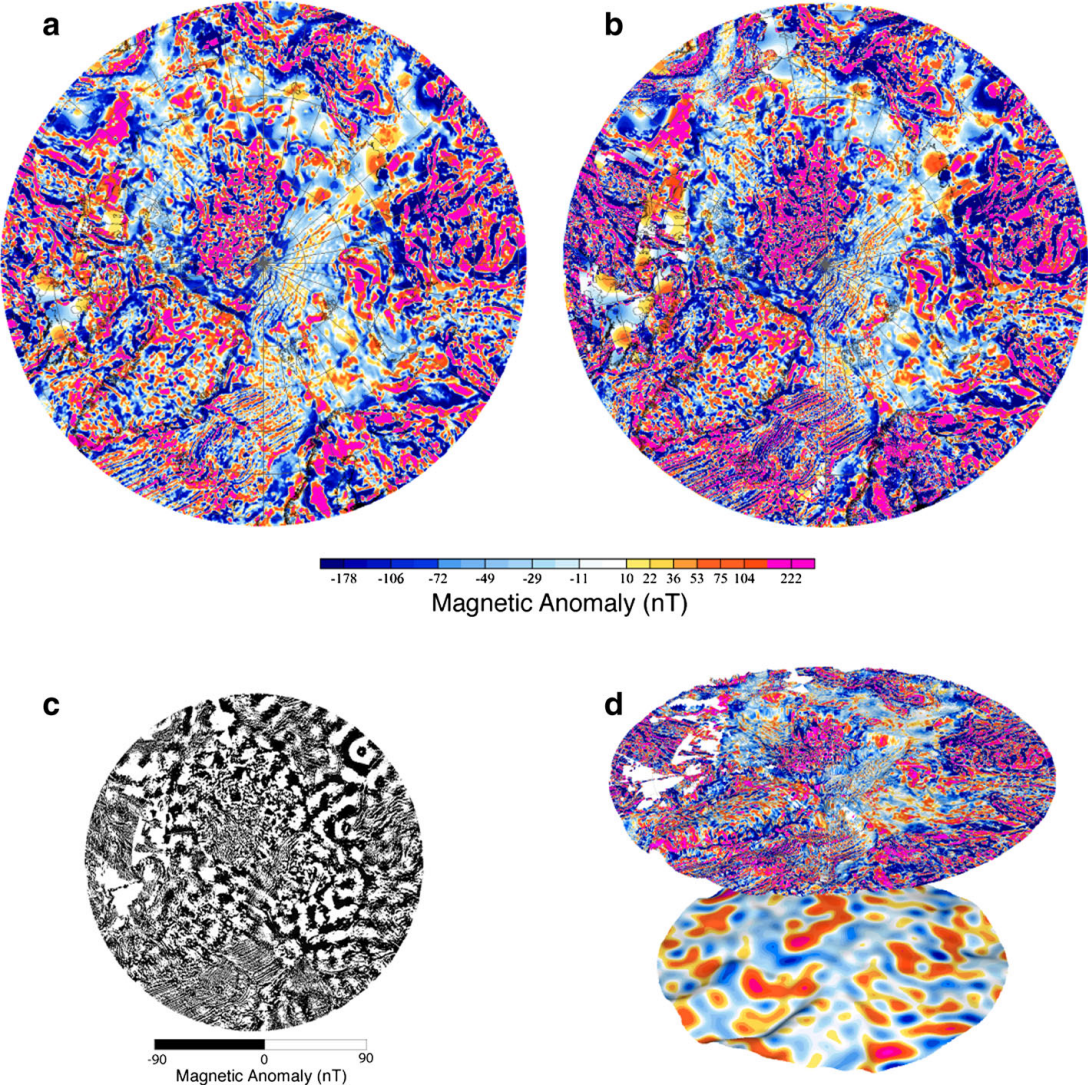
Magnetic anomaly maps: **a** WDMAM (Korhonen et al. 2007), **b** CAMPGM (Gaina et al. 2011), **c** Difference between CAMPGM and WDMAM, **d** *Top*: 3D view of CAMPGM magnetic anomaly map draped on bathymetry and topography of the Arctic (IBCAO, Jakobsson et al. 2008), *Bottom*: lithospheric magnetic model MF6 (Maus et al. 2009) downward continued at the geoid height draped on the CAMPGM Bouguer anomaly map (see text for further details)

Access to the remote sensing data over the Arctic region revolutionized the way we “see” this inaccessible territory. The high-resolution marine gravity potential fields derived from ERS-1 altimeter (e.g., Laxon and McAdoo 1994) were able to provide for the first time a comprehensive view over the High Arctic. For example, the plate boundaries that were first inferred decades in advance by using plate tectonic theory only (Heezen and Ewing 1961) are visible now as continuous, linear gravity anomalies. A new generation of regional data sets was published in the last decade—they used either the newly available satellite data or compiled major airborne, surface, icebreaker and submarine data that were combined with satellite data and models to produce maps with higher resolution than ever before (Table 1; Figs. 2, 3 and 4). The gravity and magnetic anomaly gridded data have subsequently been used to interpret the outline of main tectonic features (including the continent-ocean boundary, COB), active and extinct plate boundaries, sutures and the extent of volcanic provinces (Fig. 2e).

**Table 1:** List of gravity and magnetic digital gridded data for the Circum-Arctic region

Model	Data	Resolution	Comments	References
**Gravity**				
ArcGP *FA* (Arctic Gravity Project)	Airborne, marine, ground and submarine data	5′ × 5′ 64–90°N		Forsberg and Kenyon (2004), Kenyon et al. (2008);
CAMPGMP-GM *FA*/*BA* (Circum-Arctic Mapping Project-Gravity and Magnetic Anomaly maps)	Airborne, marine and surface data	10 × 10 km 60–90°N	EIGEN GL04C (Forste et al. 2008) for quality control of the long wavelengths	Gaina et al. (2011)
DTU10 *FA*	Satellite altimetry (ERS-2 and Envisat)	1 min	On land, the field have been augmented with the best available terrestrial gravity field complete global coverage	(Andersen, 2010)
ARCS-2 *FA* (ARCtic Satellite-only)	Satellite altimetry (Envisat and ICEsat)			McAdoo et al. (2013)
**Magnetics**				
World Digital Magnetic Anomaly Map (WDMAM)	Airborne, marine, surface and satellite data	5 km upward continued 5 km		Korhonen et al. (2007), Maus et al. (2007)
CAMPGM-GM	Airborne, marine, surface and satellite data	2 km upward continued 1 km	Downward continued lithospheric magnetic field model MF6 derived from satellite data (Maus et al. 2009) was used as a regional reference surface	Gaina et al. (2011)
EMAG2	Airborne, marine, surface and satellite data	2 min upward continued 4 km	Interpolation between sparse track lines in the oceans was improved by directional gridding and extrapolation, based on an oceanic crustal age model. The longest wavelengths (>330 km) were replaced with the latest CHAMP satellite magnetic field model MF6	Maus et al. (2009)

**Table 2:** Magnetic anomaly chrons dated by several age models and spreading rates used for synthetic model (Fig. 6)

Chron/timescale (Ma)	Channell (1995)	Ogg (1995)	Cande and Kent (1995)/Gradstein et al. (1994)	Ogg (2012)	Full spreading rate (km/Myr)
C5n old			10.949	11.056	12
C18n old			40.130	40.321	
C20n old			43.789	43.430	22
C21n old			47.906	47.329	
C24n old			53.347	53.363	
C25n young			55.904	57.101	
XR			125.500		30
M4n old	126.570				
M5n old		126.460	127.700	131.430	
M10n old	129.250	129.820	130.560	133.880	17
M11n young	130.840	131.650	132.069	135.320	
M15n young	135.570	138.300	136.675	139.590	
M16n young	136.500	141.200	137.877	140.420	10
M17n young	138.500	141.850	140.335	142.220

**Table 3:** Tomographic models showed in Figs. 9, 10, 11 and 12

Model^a^	Wave type	Reference model	Crustal model	Horizontal resolution (km)	Vertical resolution	Maximum depth (km)	References
S20RTS	S	PREM^b^	CRUST5.0^e^			CMB	Ritsema et al. (1999)
CU_SRT1.0	Surface waves	ak135^c^	CRUST5.0	200	73 layers	250	Shapiro and Ritzwoller (2002)
CU_SDT1.0	Surface waves	ak135	CRUST5.0	200	73 layers	250	Shapiro and Ritzwoller (2002)
SG06	S	PREM	CRUST5.0	275	22 layers	CMB	Simmons et al. (2006)
PRI-P05	P	iasp91^d^	CRUST2.0^f^	300–800	Not specified	CMB	Montelli et al. (2006)
PRI-S05	S	iasp91	CRUST2.0	300–800	Not specified	CMB	Montelli et al. (2006)
LH08	S and surface waves	ak135	CRUST2.0	400	16 layers	661	Lebedev and van der Hilst (2008)
MITP08	P	ak135	CRUST2.0	80	64 lyers	CMB	Li et al. (2008)
S40RTS	S	PREM	CRUST2.0	500	21 splines	CMB	Ritsema et al. (2011)
IPGG12	P	ak135	CRUST2.0		10 layers	640	Jakovlev et al. (2012)
SL2013sv	Surface waves	ak135_50	CRUST2.0	6°		660	Schaeffer and Lebedev (2013)

^a^Modified after Buiter et al. (2012)

^b^Dziewonski and Anderson (1981)

^c^Kennett et al. (1995)

^d^Kennett and Engdahl (1991)

^e^Mooney et al. (1998)

^f^Bassin et al. (2000)

To illustrate the distribution of short wavelength magnetic anomalies (which usually are produced by near-surface magnetized bodies), the CAMPGM magnetic anomaly map is also presented in a 3D view, draped on bathymetry and topography of the Arctic (IBCAO, Jakobsson et al. 2008) (Fig. 4d). The lithospheric magnetic field MF6—a model derived from satellite data and downward continued at the geoid height (Maus et al. 2009)—is draped on the Bouguer anomaly (BA) of the Circum-Arctic region. This combination of data sets aims to illustrate whether negative BA typical for areas of thick crust (mainly continental, cratonic area or large volcanic provinces) correlates with long wavelength structures of the magnetic field (which are mainly generated by deeper structures).

Gravity anomaly data can be used to estimate depth to basement and crustal thickness (e.g., Alvey et al. 2008; Minakov et al. 2012; Glebovsky et al. 2013), but additional accurate information is needed (topography, bathymetry and sediment thickness) for generating reliable models. Recently, the GOCE satellite gravity gradient data are being tested for deriving global Moho maps (Reguzzoni and Sampietro 2012)—an attempt to overcome the imperfections of the widely used CRUST2.0 model (Laske and Masters 1997; Bassin et al. 2000) that is not able to reconcile the seismological-derived crustal architecture with the observed gravity. However, this is a very simplified and coarse model (shown in Fig. 3 together with the new CRUST1.0 model—an updated and improved model of CRUST2.0), and further attempts to improve it are underway (e.g., GEMMA Project, http://gocedata.como.polimi.it). The resolution of the CRUST1.0 model is 1 degree, while the resolution of the GEMMA model is 0.5 degrees. Note that the depth to Moho presented by the two models shows differences up to ±20 km.

Some of the global and regional maps are regularly updated, especially with improved satellite data and any new available surface data (e.g., the DTU gravity anomaly map). In the case of magnetic anomaly compilations, international efforts for sharing data and to contribute to regional maps have proved extremely useful [like the WDMAM and CAMP-GM initiatives; (Korhonen et al. 2007; Maus et al. 2007; Gaina et al. 2011)]. Hopefully, the wealth of data that has been acquired under the Law of the Sea in the last couple of years will contribute to improve the existing geological and geophysical data sets of the Arctic region and will set the scene for more detailed “views” of the Arctic’s structure.

Besides new gravity and magnetic compilations and models (few examples shown in Figs. 2, 4), an updated bathymetric grid (Jakobsson et al. 2012), and an improved compilation of crustal and sediment thickness (Laske et al. 2013) form the basis for regional studies on tectonic evolution of this remote area. The sediment thickness data set CRUST2.0 (Laske and Masters 1997; Bassin et al. 2000) that covers the Arctic region was obtained by digitizing the Tectonic Map of the World provided by the EXXON production research group (1985). CRUST1.0 improves the global sediment thickness database only with some near-coastal new data, but not in the Arctic region. Note that local and regional maps of sediments thickness that postdates the EXXON database have been produced by compiling sediment thickness interpreted from 2D seismic data (e.g., Gramberg et al. 1999), or indirectly by using the inferred depth to (magnetic) basement from magnetic data interpretation (e.g., Glebovsky et al. 2003), and more recently by compiling vintage and recent data from seismic transects (Kashubin et al. 2012) as a contribution to the new Tectonic Map of the Circum-Arctic region (Petrov et al. 2013).

**Fig. 5:**
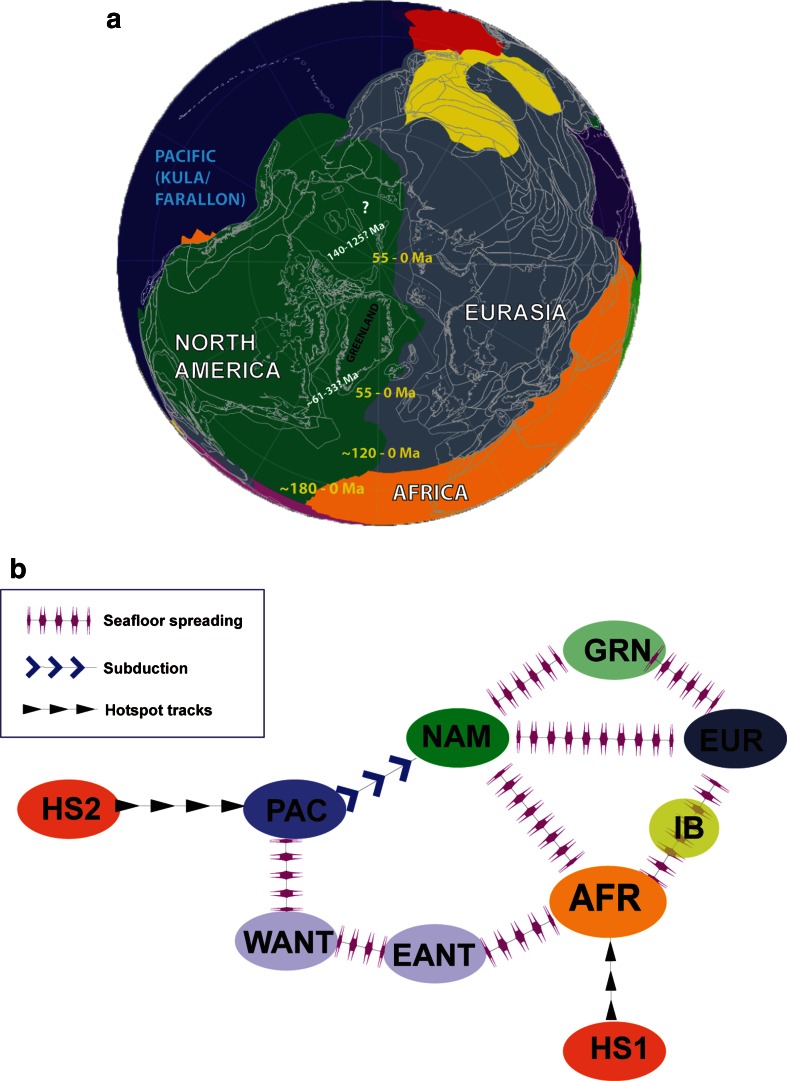
**a** Present-day outlines of the Circum-Arctic tectonic features used for reconstructions in this paper. Numbers in yellow and white indicate the age of North Atlantic and Arctic. **b** Simplified relative plate motion hierarchy used in computing the hotspot motion track in the Circum-Arctic region. *EANT* East Antarctica, *EUR* Eurasia, *GRN* Greenland, *HS* hotspot, *IB* Iberia, *NAM* North America, *PAC* Pacific

Numerous other, local or regional, geophysical and geological data (including seismic reflection and refraction, IODP, ODP, piston cores and dredge data) together with global data and models (like seismic tomography) can advance our understanding of how basins formed and how continental masses dispersed and accreted. Although none of the regional data sets contain information that alone will shed light to the formation of the High Arctic, we here present a review of the structure and kinematic hypotheses that are inferred from available data and discussed in the recent literature. Tectonic boundaries derived from the Arctic geophysical and geological data (like the one presented in Fig. 2e) and other regional tectonic features described in the literature (e.g., Alvey et al. 2008; Torsvik et al. 2010) are schematically shown in Fig. 5a.

## Oceanic Versus Continental Crust and Magmatic Activity in the Arctic Region

### Amerasian Basin

#### Canada Basin

**Fig. 6:**
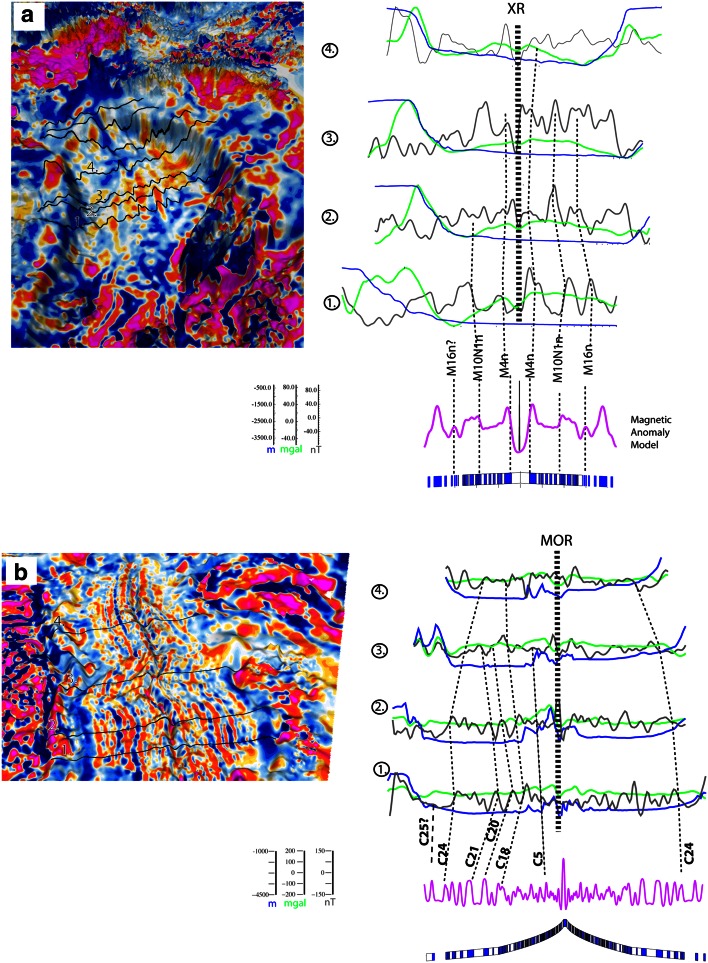
Seafloor spreading magnetic anomalies in the Canada (**a**) and Eurasia (**b**) basins. Figures on the left show gridded magnetic anomalies from the CAMPGM map (Gaina et al. 2011), and on the right selected profiles perpendicular on direction of seafloor spreading of observed magnetic anomaly (in *black*), gravity anomaly (in *green*) and bathymetric data (in *blue*). Synthetic magnetic profiles (in *magenta*) modelled by using a series of normal and reversed polarized bodies (shown as *blue* and *white* *series of* *rectangles*). The Cenozoic magnetic chrons identified in the Eurasia Basin are numbered from C5 to C25, and the Mesozoic chrons in the Canada Basin are numbered from M4 to M16 (see Table 2 for chron ages). MOR is active mid-ocean ridge and XR is extinct ridge

Situated between the Canadian and Alaskan margins, this almost triangular-shaped deep basin (Fig. 1) is characterized by linear and apparently fan-shaped magnetic anomalies (Figs. 4, 6) and by a curved gravity anomaly that presumably represents an extinct mid-ocean ridge (Fig. 2). Although this basin was less affected by volcanism in comparison with other parts of the Amerasian Basin, very thick sediment cover and a complex magnetic anomaly pattern make it difficult to establish the age and nature of this basin. Several magnetic profiles extracted from the CAMP-GM magnetic grid are shown together with a synthetic model that suggests an Early Cretaceous age, probably M4n to M10N (Fig. 6a; Table 2). Although some of the magnetic anomalies that flank the interpreted extinct ridge could be of Mesozoic age (Fig. 6a), the asymmetry of the basin flanks, its magnetic anomaly pattern and increasing evidence from seismic data point now to a more complex structure of this basin, and it has been postulated that more than half of the basin’s basement is (extended) continental and transitional crust (possibly exhumed subcontinental mantle, Grantz et al. 2011; Mosher et al. 2012).

The most commonly accepted model for explaining the opening of the Canada Basin includes counterclockwise rotation (CCR) of Arctic Alaska away from the Canadian Arctic islands (Carey 1955). An alternative model was proposed by Koulakov et al. (2013) and presumes the clockwise rotation of central Arctic (Arctida Block) due to subduction of the oceanic lithosphere underneath the Anyui Suture. A more unconventional model postulated that this basin formed in North Pacific and was subsequently trapped in the Arctic region (Churkin and Trexler 1980).

The rotational model is based on paleomagnetic data (Halgedahl and Jarrard 1987), stratigraphic studies of the North Slope and Sverdrup Basin margins, and a fan-shaped magnetic pattern observed in the southern Canada Basin. Both stratigraphic studies (e.g., Embry 1990 and references therein) and magnetic anomaly patterns have been the subject of numerous discussions and reinterpretations. Lane (1997) and Grantz et al. (1998, 2011) proposed more complex models that include orthogonal or strike-slip motion, combined with a rotation in the later stages of opening.

**Fig. 7:**
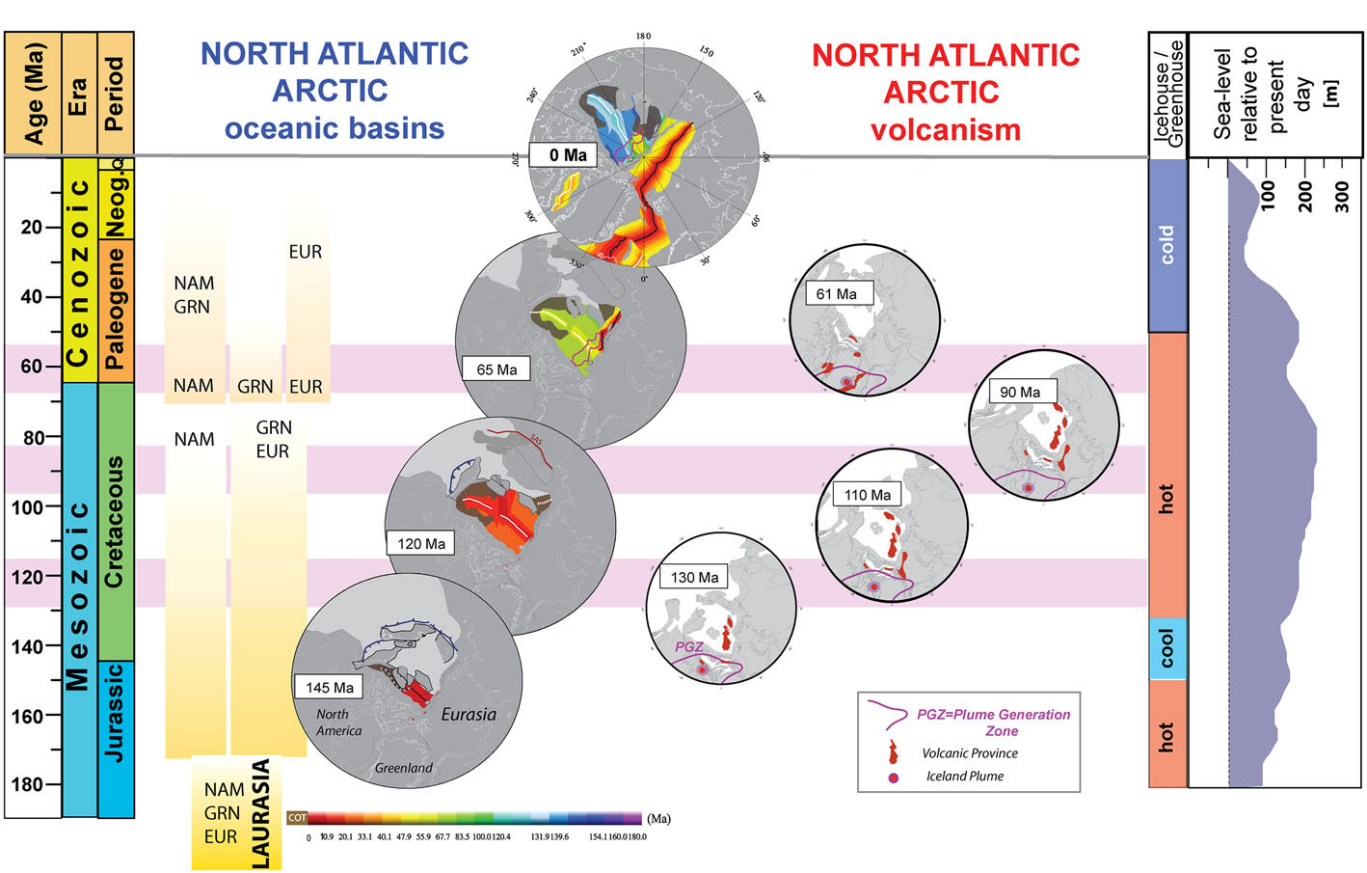
Overview of the evolution of the Arctic region with an emphasis on the opening of main oceanic basins and volcanic episodes. Inferred volcanic periods are shown as *horizontal pink bars*. First-order sea-level changes and main climate regimes are also shown

Using a new interpretation for the South Anyui Suture (SAS, see Fig. 2) that was formed when an older Arctic oceanic basin—the South Anyui—closed as a result of terrane docking with the Siberian craton and Eurasian margin, (Kuzmichev 2009) proposed a “two-pole parallelogram hypothesis” (or “saloon door” model) to link the Amerasia Basin formation due to northward subduction under the Alaskan North Slope and Chukotka. A slightly modified version of this model is shown here for explaining extension and seafloor spreading in the Canada Basin (and by inference north of it, and possibly in the Alpha Ridge region as well) (Fig. 7).

#### Northwind Ridge and Chukchi Plateau

These submerged rifted plateaus are located north of the Chukchi Sea (Fig. 1) and are identified to be of continental nature. Data from cores collected from the southern Northwind Ridge show that Triassic and older strata were attached to both Arctic Canada and Alaska prior to the rifting that created the Canada Basin, but controversies exist in establishing the conjugate margins (for a review see Grantz et al. 2011). Younger sediments show that this continental sliver was isolated in the Early Jurassic (Grantz et al. 1998). Northwind Ridge was uplifted later (in the Paleocene), perhaps due to relative convergence with other adjacent tectonic blocks, while extension relative to the Chukchi Borderland created the Northwind Basin.

#### Alpha and Mendeleev (AMR)

The nature of both the Alpha and Mendeleev Ridges remains speculative because extensive volcanism has overprinted and complicated the original geophysical signatures. Maastrichtian fossils have been recovered (Clark et al. 1980), and sediments from the cores (Late Campanian to Early-Maastrichtian, 76–69 Ma, Davies et al. 2009) provide a minimum age constraint. Volcanic material, 82–72 Ma in age, has been described from the Alpha Ridge (Jokat 2003), and the similarity with the Iceland–Faeroes plateau led Vogt et al. (1979), Lawver et al. (1983) and Forsyth et al. (1986) to suggest an “hotspot-related” origin. Aiming to clarify the origin and structure of basement and sediment cover of Alpha and Mendeleev Ridges, a wealth of geophysical data and piston core samples has been acquired in the past few years. A continental-like intruded crust has been suggested by Kaminsky (2005), an oceanic origin by Jokat (2003), and undecided (rifted volcanic continental margin or oceanic plateaus formed at spreading centres) by Dove et al. (2010). Some authors (e.g., Filatova and Khain 2010) propose that these ridges might be the remnant parts of a larger continental plate which existed in the Arctic and was destroyed due to different tectonic episodes. Recent seismic studies have provided evidence for continental crust (about 32 km thick) underlying at least part of the Mendeleev Ridge (Lebedeva-Ivanova et al. 2006). Bruvoll et al. (2012) concluded that the upper volcanic carapace of Mendeleev and north-western Alpha Ridge were most likely emplaced during a brief igneous episode—no later than Campanian (80 Ma)—as part of the latest events of the Late Cretaceous Circum-Arctic volcanism.

New high-resolution magnetic anomaly data collected along the Alpha Ridge, situated near the southern Lomonosov Ridge and Greenland, revealed dyke swarm-like linear magnetic anomalies that resemble anomalies identified in Ellesmere Island and Franz Josef Land (Døssing et al. 2013). These authors concluded that part of the Alpha Ridge is highly attenuated continental crust formed by poly-phase break-up with LIP volcanic addition, but they also interpreted Barremian (or alternatively lower Valanginian–Barremian) seafloor spreading anomalies in the Makarov Basin region.

#### Lomonosov Ridge

The western boundary of the Eurasian margin, the Lomonosov Ridge, indisputably rifted away from the northern Barents Sea during the Paleocene as a narrow microcontinent (ca. 55 Ma, e.g., Srivastava 1985). Its continental nature was recognized from seismic data that showed horsts and grabens (e.g., Jokat et al. 1992), and it is the only Arctic location drilled under the IODP programme (IODP Expedition 302, 2006). Recent investigations have shown a more complex nature with detailed pull-apart basins in its central part, a volcanic plateau close to Greenland, and extended crust at its opposite end, close to the East Siberian Shelf (e.g., Lebedeva-Ivanova et al. 2006; Jackson et al. 2010).

#### Podvodnikov and Makarov Basins

The Podvodnikov Basin occupies the space between Mendeleev Ridge, the East Siberian Shelf and the Lomonosov Ridge. The Makarov Basin is much deeper, with its northward continuation bordered only by the Lomonosov and Alpha Ridges. Seismic reflection and refraction studies reveal a thicker oceanic crust for the Makarov Basin (~22 km) and part of the Podvodnikov Basin (~20 km) (Sorokin et al. 1999; Lebedeva-Ivanova et al. 2006). Regarding the formation of these basins, one end-member hypothesis considers the Makarov Basin as the continuation of the Canada Basin, thus implying a similar age and structure. Recently, Døssing et al. (2013) interpreted N–S magnetic lineations as chrons M16n–11An.1n (Early Valanginian–Late Hauteverian, 138–132 Ma) and chrons M10n/9n–4n (Barremian, ca. 129–126 Ma). Alternatively, a Late Cretaceous–Early Tertiary age has been postulated. Weber (1990) suggested an age between 118 and 56 Ma, and Mickey (1998) an age of 95 to 67 Ma.

These uncertainties led Alvey et al. (2008) to examine three new plate tectonic scenarios for the age and opening of the Makarov and Podvodnikov Basins, in which they assigned an Early- to Mid-Cretaceous age to the Canada Basin and varied the age and the crustal nature between (and within) the Alpha Ridge and Mendeleev and Lomonosov Ridges. This range of oceanic age distributions has been used to determine the crustal thickness of these basins, and the results were compared with the estimates from refraction data modelling. They concluded that the Podvodnikov Basin is probably Late Cretaceous in age. Our interpretation is that these basins, whether they are floored by oceanic or extended continental crust, are the result of the Late Cretaceous–Cenozoic extension between the North America and Eurasia, as predicted by the regional model of Gaina et al. (2002). New seismic profiles acquired across the Lomonosov Ridge and adjacent basins interpreted by Langinen et al. (2009) have confirmed that the Marvin Spur is a sliver of continental crust (as also suggested by Cochran et al. 2006) and that part of the Makarov Basin probably formed in the Early Tertiary on thinned continental crust.

### Eurasian Basin

Well-preserved linear magnetic anomalies (isochrons) that are relatively easy to identify have allowed a straightforward interpretation of the Eurasian Basin (Figs. 4, 6b). Most authors have identified chron 24 (ca. 54 Ma) as the oldest magnetic isochron, spawned by seafloor spreading between the Lomonosov Ridge and the Eurasian margin (Srivastava 1985; Gaina et al. 2002; Glebovsky et al. 2006). Other studies have identified an abandoned extinct ridge (ca. 55 Ma) in the proximity of Lomonosov Ridge (Brozena et al. 2003). If correct, this structure implies that the opening of the Eurasian Basin was linked to the evolution of Baffin Bay and the Labrador Sea, but the restoration of this plate boundary is made difficult by the subsequent Eurekan compressional phase. New studies suggest that the previously interpreted chron 25 (ca. 56 Ma) may represent serpentinized exhumed mantle formed before break-up together with the highly thinned continental crust that can be observed only on the Lomonosov Ridge conjugate margin (Minakov et al. 2012). The age of the oldest oceanic crust in our Eurasia Basin model (Fig. 6) is taken as 56 Ma (C25).

### Cretaceous to Present Magmatic Activity in the Arctic

Scattered magmatic areas have been identified in the Arctic along the continental margins (North Greenland, East Siberian Islands, Svalbard, Franz Josef Land, e.g., Maher 2001; Buchan and Ernst 2006; Kuzmichev et al. 2009; Tegner et al. 2011; Corfu et al. 2013; Nejbert et al. 2011) on submerged microcontinents (Chukchi Plateau) and submarine plateaus of unknown or controversial nature (Alpha and Mendeleev Ridges, Yermak and Morris Jesup Plateaus, e.g., Jokat et al. 1995 and 2008) and within the extended continental crust (e.g., Barents Sea, Minakov et al. 2012). Volcanism spanning from about 130 to 60 Ma probably occurred during at least two phases—an initial tholeiitic phase (130–80 Ma) and a second alkaline phase (85–60 Ma) (for a review see Tegner et al. 2011) and is labelled as the High Arctic Large Igneous Province (HALIP) in the literature (see Buchan and Ernst (2006) for a review). The tholeiitic phase was probably the result of mantle plume activity.

Assuming that the Iceland plume was active since the Cretaceous, its restored Early Cretaceous position is located in the Arctic region, as suggested in the early 1980s by Morgan (1983). The Lawver and Müller (1994) classical paper showed that the computed Iceland plume position at 130 Ma assuming a fixed hotspot reference frame (Müller et al. 1993) was very close to the presumably massive Early Cretaceous volcanism on the Alpha Ridge, as also suggested by Forsyth et al. (1986).

**Fig. 8:**
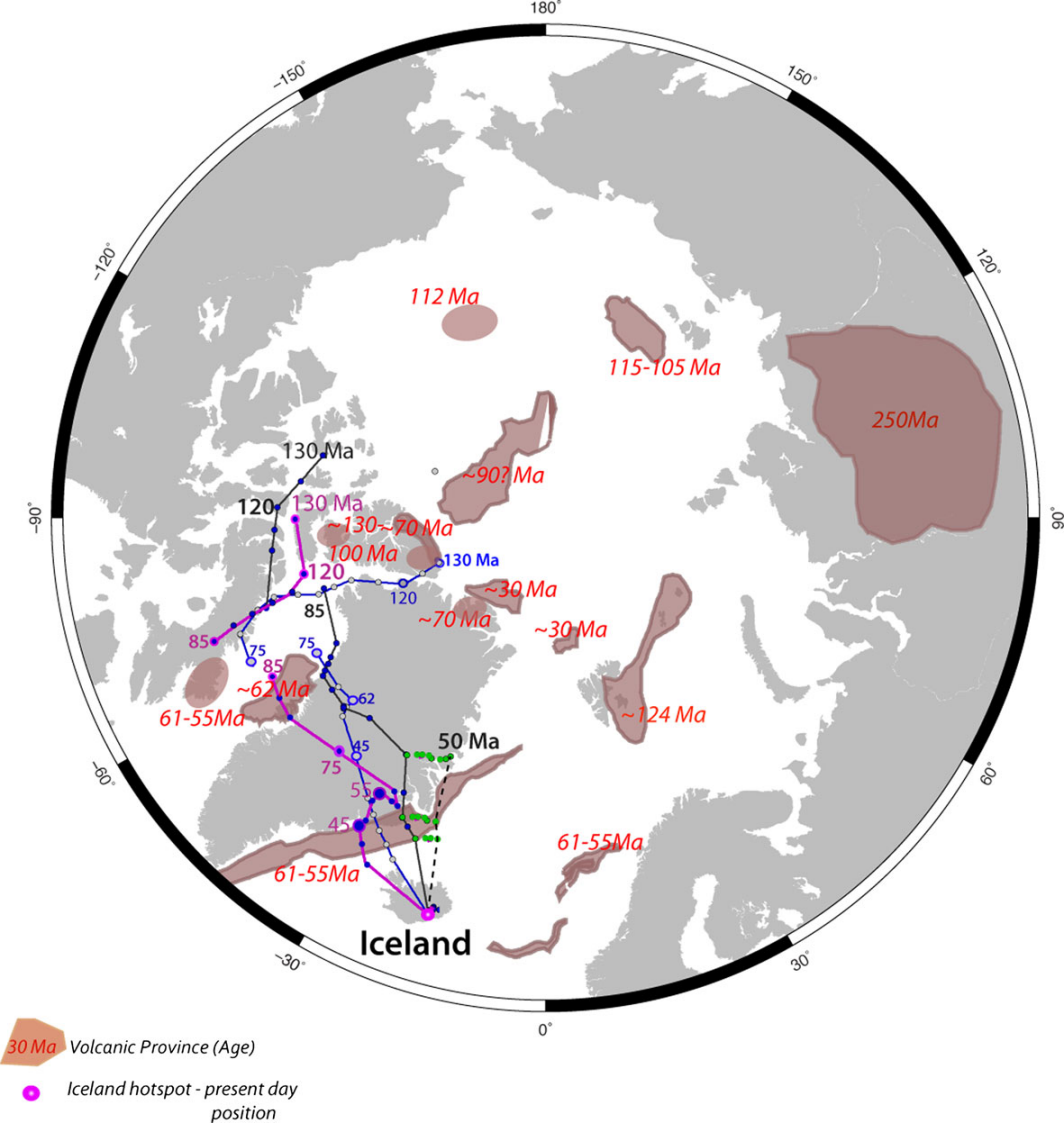
Iceland hotspot tracks according to the Lawver and Müller (1994) model (*blue path*), Torsvik et al. (2008) model (*black track*) and Doubrovine et al. (2012) model (*magenta track*); the hotspot position is fixed to the mantle. The restored hotspot track for the last 50 million years with a correction for the hotspot tilting is shown by the *dashed line* (and the *green dots* that indicate the amount of displacement from the curve calculated for a stationary plume). We have corrected only the hotspot track modelled with the Torsvik et al. (2008) reference frame

The fixity of hotspots relative to the mantle has since been disputed, and it has been shown that, due to mantle advection, hotspots are tilted, and therefore, their “surface-projected” position is changing through time (Steinberger and OConnell 1998). In this paper, we show two alternative models for the Iceland plume path by using updated global tectonic plate absolute and relative plate circuits (Fig. 8). One model is computing the absolute motion of Africa relative to moving hotspots based on Indo-Atlantic hotspot tracks and mantle convection models for the past 100 million years (Torsvik et al. 2008). For older times, a longitude-adjusted paleomagnetic reference frame was considered. A new global reference frame model (Doubrovine et al. 2012) uses relative plate motions based on paleomagnetic data and marine geophysical data plus estimation of both Pacific and Indo-Atlantic hotspot motion relative to the mantle to construct a self-consistent global model for relative and absolute motions for the past 124 Ma. This model has been merged with a true-polar wander (TPW) corrected paleomagnetic reference frame before 124 Ma.

Compared to the fixed hotspot model (Lawver and Müller 1994), the combined moving hotspot- and paleomagnetic-based models (Torsvik et al. 2008 and Doubrovine et al. 2012) predict the Early–Mid-Cretaceous position for the Iceland hotspot in the western and central Ellesmere Islands, respectively, more than 1,000 km away from the previously inferred location of a fixed mantle plume (Fig. 8). The new predicted hotspot tracks are farther away from the Alpha and Mendeleev Ridges, but closer to the Axel-Heiberg Island dykes dated by the ^40^Ar–^39^Ar method as 128.2 ± 2.1 Ma (Villeneuve and Williamson 2003).

Possible links to other HALIP volcanic regions can be better visualized on maps showing the position of tectonic blocks in an absolute reference frame, and we have computed plate reconstruction models for 130, 110, 90 and 61 Ma that may represent the more intense magmatic periods reflected by the ages of volcanism in Greenland, Ellesmere Islands, Svalbard, Franz Josef Land and possibly New Siberian Island, Chukchi, and Alpha–Mendeleev Ridges (Fig. 7). In addition, the so-called Plume Generation Zones (PGZs)—regions at the core–mantle boundary where plumes initiate and eventually generate large igneous provinces and surface hotspot volcanism through time (e.g., Torsvik et al. 2006)—is also shown on these reconstructions. Arctic magmatic provinces may be related and generated from a PGZ from ca. 130 to 100 Ma, as North Greenland, Ellesemere Islands, Svalbard, Franz Josef Land and possibly Alpha Ridge were in the proximity of its northernmost prolongation, but ~115–80 Ma magmatism on the Chukchi Plateau, New Siberian Island and Mendeleev Ridges is probably not related to it, unless it can be shown that the PGZ-generated volcanism extends on very large areas (with a radius of more than ca. 1,000 km). In Paleocene time, the northern PGZ coincides well with the NAIP volcanism, including the Disko Island (~62 Ma, Storey et al. 1998) magmatism that cannot be well explained by the new Iceland track locations based on the moving hotspot absolute reference frames of Torsvik et al. 2008 and Doubrovine et al. 2012 (Figs. 7, 8).

## Circum-Arctic Mantle Imaged by Tomography Models

The structure of the mantle in the Arctic region has rarely been discussed in studies dedicated to global tomographic models, either because of the lack of proper seismic ray path coverage or because of a lack of interest in this important area. Several studies present adjacent areas like the North Pacific (e.g., Gorbatov et al. 2002) and North Atlantic (e.g., Rickers et al. 2013) where subduction processes and mantle plumes may have generated interesting structures prone to be imaged by seismology. Exception to this are studies of Levshin et al. (2001) and Jakovlev et al. (2012). Fortunately, in the last decade, due to the availability of much improved global earthquake catalogue data that can resolve the northern higher-latitude area, increased resolution global and regional models are now able to shed light on the upper and lower structures of the mantle.

**Fig. 9:**
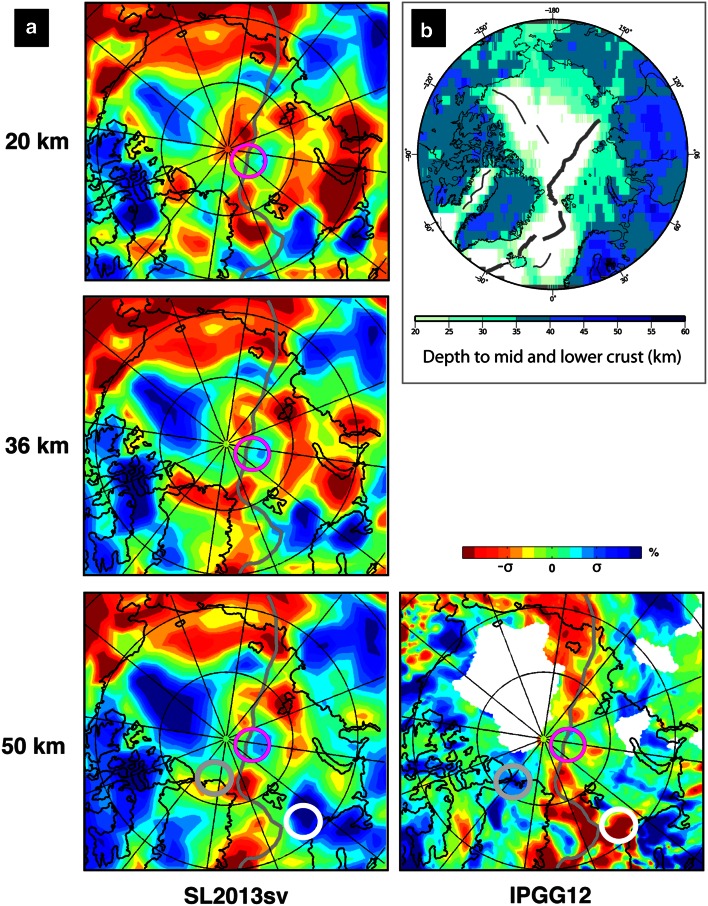
**a** Global (SL2013) and regional (IPGG12) tomographic models at shallow depths in the Arctic region. For easy comparison between models, we scale the tomographic models individually using standard deviation (sigma) calculated for each depth interval. This way, the colour intensity is similar for all the models, even though the anomaly absolute value differs significantly, from sigma = 0.9 % (IPGG12 model at depth of 50 km) to sigma = 2.6 % (SL2013 at 20 km). See Table 3 for tomographic models description. **b** Depth to mid- and lower crystalline crust (CRUST1.0 model). *Thick grey lines* indicate active mid-ocean ridges, and *thin lines* are inactive plate boundaries. See text for discussions

We have chosen several global models and one regional model that image the whole mantle and the upper mantle, respectively, published from 1999 to 2013, and which are publicly available (Table 3), to extract vertical and horizontal cross-sections. Shallow horizontal cross-sections through the lower crust and upper mantle (at 20, 36 and 50 km) are shown in Fig. 9. Both tomographic models (SL2013sv by Schaeffer and Lebedev (2013) and IPGG12 by Jakovlev et al. (2012)) used CRUST2.0 model to correct for crustal thickness, but Schaeffer and Lebedev (2013) included shallower nodes (at 7, 20 and 36 km) in their inversion to minimize the inaccuracies of CRUST2.0. Excluding the area with very poor data coverage (shown as a white patch on the IPGG12 model map), there are a few similarities, but also some differences, in the two models. One area that seems to be consistently imaged by both models is the Gakkel Ridge with low velocities at both ends and relatively higher velocity in the middle (see the magenta circle in Fig. 9). It is well known that this is the slowest spreading ridge in the world, and it has been postulated that it is formed by mostly amagmatic spreading processes, and has very thin crust (2–4 km, e.g., Jokat and Schmidt-Aursch 2007; Michael et al. 2003). However, studies based on a new, local seismological data set suggest that thicker crust (~7 km) has been produced along the central Gakkel Ridge and earthquake hypocentre depths reached up to 22 km below the seafloor (Schlindwein 2013). These observations together with the segmented pattern observed in the shallow tomographic models (Fig. 9) are likely to provide more insight into the structure and the processes of present-day plate boundaries along the Eurasia Basin.

In contrast, the two models seem to differ in imaging the crust of the SW Barents Sea, with the SL2013sv model showing large velocities (standing presumably for thick crust) and the IPGG12 model showing small velocities (Fig. 9—area indicated by white circles). Conversely, the region of northern Greenland has small velocities in the SL2013sv model and large velocities in the IPGG12 model (Fig. 9—area indicated by grey circles). Both these regions are at the boundary between thick cratonic crust and thinner crust of sedimentary basins and, at the same time, closer to active mid-ocean ridges; therefore, more information from improved tomographic and crustal studies will help understand the plate boundary evolution processes. It should be noted that the IPGG12 model provides the P-velocity anomalies, whereas SL2013sv images the S-anomalies. Also, at 50 km depth, the ray paths of body waves used in the IPGG12 model in most areas do not form a sufficient intersection system; thus, the result might represent averaged values of crustal and mantle anomalies down to 100 km depth. The differences observed in Fig. 9 can be partly explained by these two reasons.

**Fig. 10:**
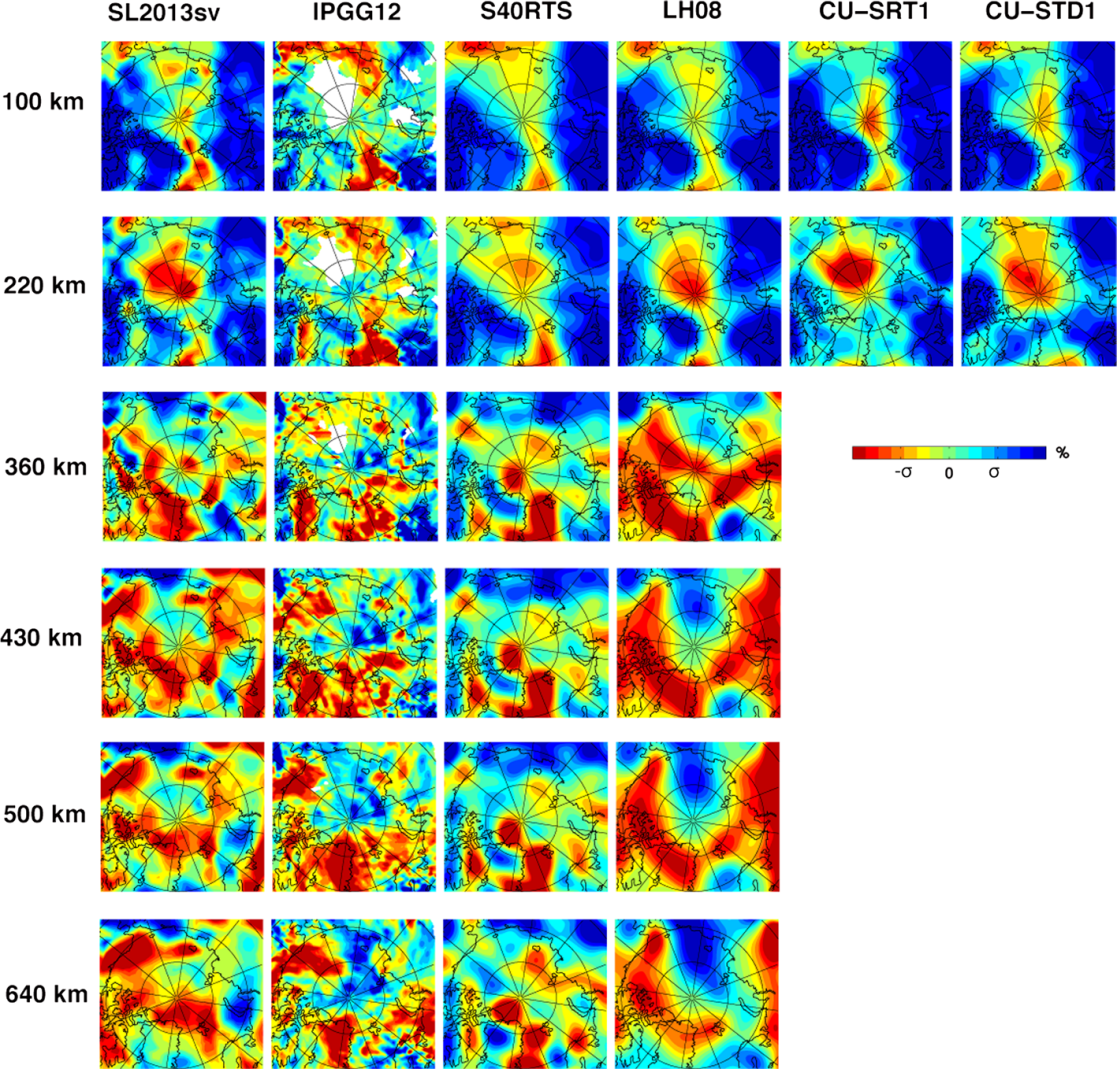
Global and regional upper mantle tomographic models—*horizontal slices* at selected depths in the Arctic region. See Fig. 9 for explanations of the colour scheme

**Fig. 11:**
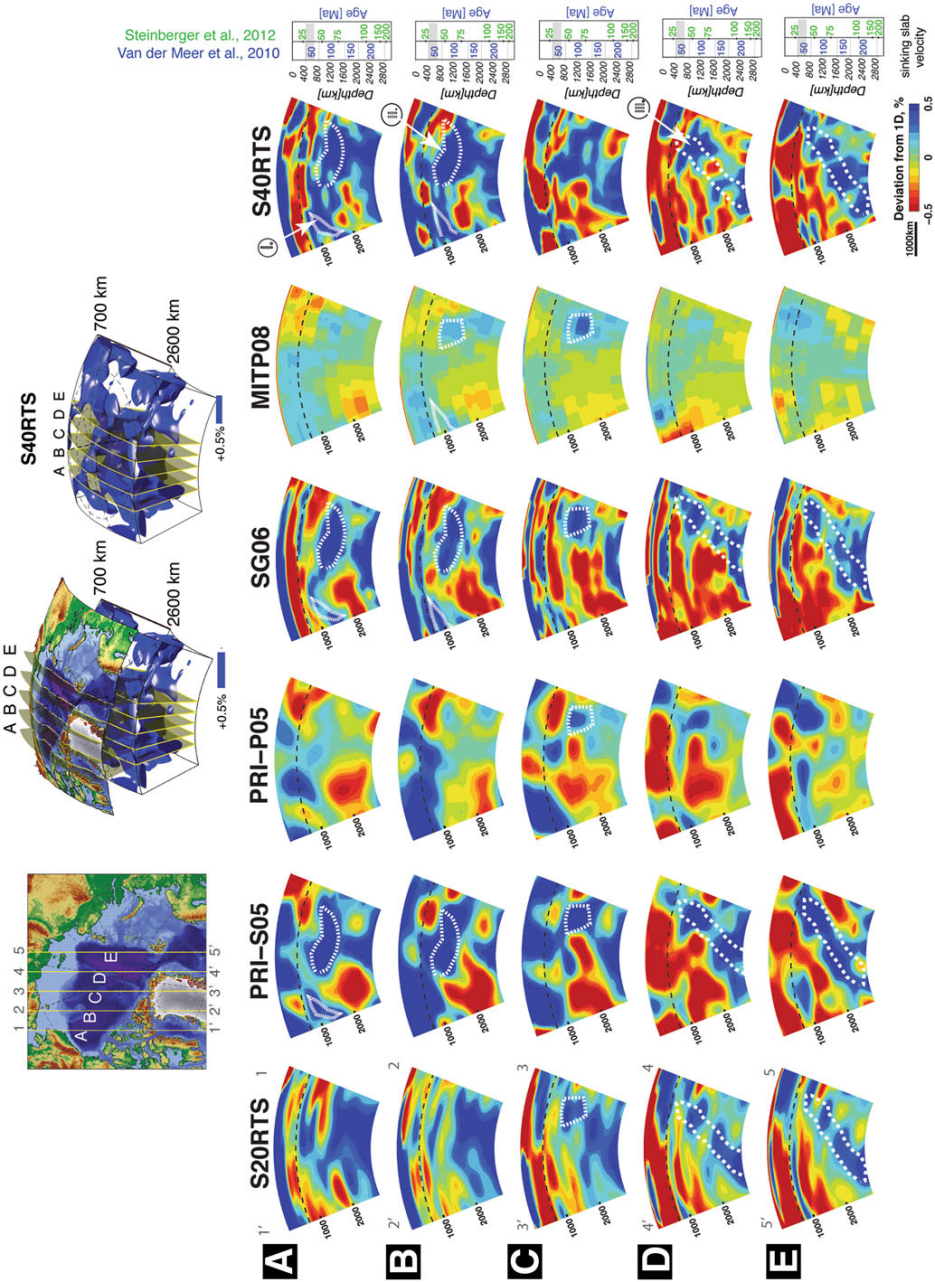
Global, whole mantle tomographic models—cross-sections along selected profiles in the High Arctic. The *dashed line* on cross-sections shows the 660 km seismic discontinuity (roughly indicating the boundary between the upper and lower mantle). See text for discussion on lower mantle structure in various models and profiles

A range of additional tomographic models is presented for the upper mantle (Fig. 10). The models of Lebedev and Van der Hilst (2008)—LH08 and Shapiro and Ritzwoller (2002)—CU-SRT1 and CU-SDT1 (that are only imaging the upper 220 km) mainly show the hot mantle under the active North Atlantic and Eurasian Basin mid-ocean ridges, and the cold cratonic areas of the North American, Eurasian and Greenland continents. In contrast, the SL2013sv (Schaeffer and Lebedev 2013) and IPGG12 (Jakovlev et al. 2012) models indicate more variations in the continental area—for example, a thicker (and colder) North Greenland and a hotter region under the North Barents Sea. While the LH08 model images a region of cold mantle under the Northwind Ridge–Chukchi Plateau, Wrangel Island region from 360 km to the transition zone (640 km), the IPGG12 model indicates two smaller regions of cold mantle closer to the North Pole and under the New Siberian Islands in the lower upper mantle (Fig. 10). The latter features are also present in the whole mantle tomographic model of Simmons et al. (2006) (Fig. 11, profiles B–E). The upper mantle imaged by the whole mantle global model S40RTS (Ritsema et al. 2011) shows a strong negative (presumable hotter) mantle anomaly north of Greenland (and the same is observed in the SL2013sv model).

Upper mantle imaging strongly depends on the crustal model used, and the interpretation of anomalies is hampered by the complexity of the mantle structure, temperature, chemistry and anisotropy (e.g., Bastow 2012). Therefore, besides presenting the available models for the Arctic region upper mantle, we will restrain from further interpretation of these models.

The lower mantle is probably more robustly imaged, and we may use these models to decipher the earlier evolution (before the development of preserved oceanic basins) of the Arctic region. We have selected a range of global models aiming to illustrate how additional data and techniques have improved the quality of the High Arctic lower mantle images. More publicly available global models were scrutinized, but some of them presented seriously “smeared” structures and were not selected here. Vertical cross-sections along five profiles that run from the North American–Greenland margins to the Eurasian margin were extracted from six tomographic models (Table 3).

All cross-sections remarkably show three groups of positive mantle anomalies: one composed of a sub-horizontal or slightly southward-dipping feature, around 1,000 km depth observed on profiles A and B (I in Fig. 11), another sub-horizontal anomaly at around 1,500 km is seen on profiles A, B and C (II in Fig. 11), and another steeper, dipping northward segmented feature starts below 1,000 km and is reaching the core–mantle boundary (CMB) and is seen on profiles B to E (III in Fig. 11). 3D images of isosurfaces showing these positive anomalies (possibly representing cold subducted material) are also shown in Fig. 11 to better illustrate the extent of the “slab graveyard” under the High Arctic region.

We attempt to interpret the nature and age of these anomalies by (1) applying an age–depth relationship and (2) analysing kinematic models of the Arctic. Several studies are now suggesting that the depth of positive anomalies observed in the mantle tomographic models could be roughly correlated with the age of assumed subducted oceanic slabs. This link was established by a series of observations and models, and several simple assumptions have to be imposed in order to achieve a very nascent linear relationship. We use two models that suggest depth–age relationship for interpreted subducted slabs: van der Meer et al. (2010) and Steinberger et al. (2012) (Fig. 11). According to these models, the three groups of sinking slabs may have ages between 85 and 95 (55) for group I, around 130 (80) for group II, and between 100 and 200 (75–200) for group III (ages according to Steinberger et al. (2012) are in italic) (Fig. 11).

**Fig. 12:**
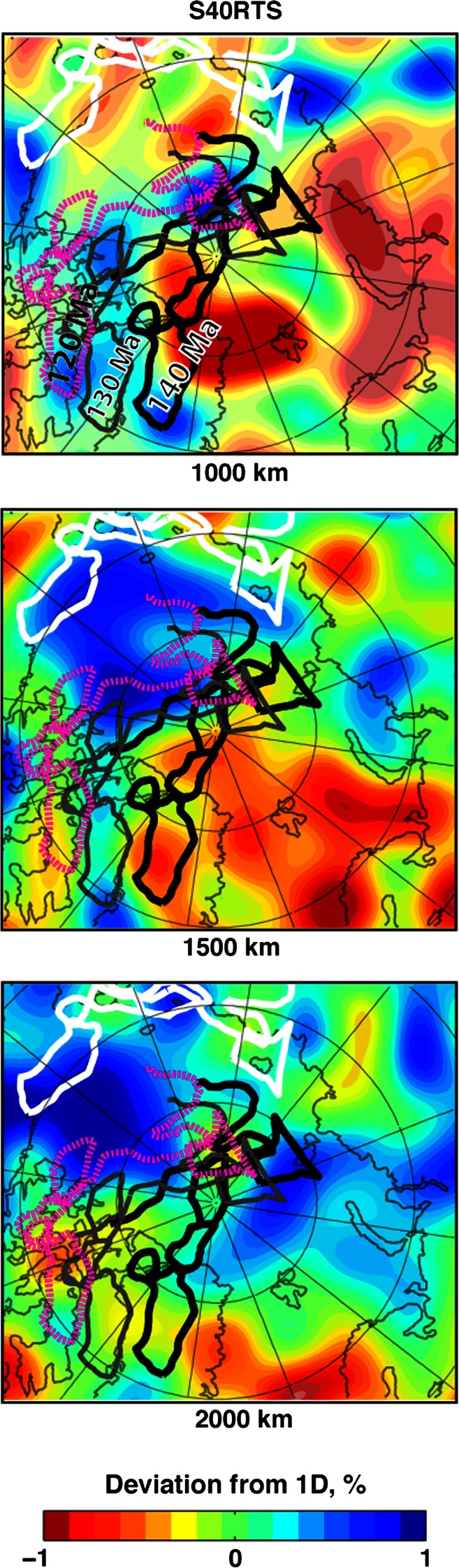
Horizontal slices at 1,000, 1,500 and 2,000 km through the global mantle model of Ritsema et al. (2011) showing the accumulation of subducted slabs at high latitude north of Alaska and north of the Barents and Kara Seas. The present day (*white contours*) and reconstructed South Anyui Suture, Chukotka Block, New Siberian Island block and the Alaska North Slope are shown at 120, 130 and 140 Ma

Before the opening of the Canada Basin, an older oceanic basin, the South Anyui Basin, occupied the area between the North American and North Eurasian margins. This basin was gradually consumed by subduction along the South Anyui (North Siberia) subduction zone until the Chukotka plate in NE Russia collided with Siberia (e.g., Sokolov et al. 2002) at around 118–120 Ma. If the subduction was oriented southward, as proposed by Koulakov et al. (2013), then the slab may have travelled toward the north-east Asia region, and a zone of fast seismic velocities immediately south of the South Anyui Suture (Fig. 2) could be the remnants of the South Anyui Ocean. However, the collision along the South Anyui Suture occured at around 120 Ma when the Chukotka and associated terranes were situated closer to the North Pole in our tectonic model, in which case the slabs might have been trapped at more northerly latitudes (Fig. 12). This model explains the pervasive group of positive mantle anomalies that are located in the lower mantle below 1,000–1,200 km and continue down to the core–mantle boundary. Note that Koulakov et al. (2013) postulated that the remnants of the South Anyui Ocean subducted slabs may be imaged by the high-velocity anomalies in the upper mantle just above the transition zone observed in the IPGG12 model, but we would argue that a different sinking mechanisms and rates are required for this interpretation.

As Kuzmichev (2009) suggests, the South Anyui subduction zone was a continuation of the trench situated south of the Arctic Alaska and associated accreted terranes (that latter became the Angaucham Suture, see Nokleberg et al. 1998). His “bipolar opening” of the Arctic Ocean as a back-arc basin driven by a northerly directed subduction zone seems to agree with the shallower slab remnants imaged by seismic tomography under the Arctic region (Figs. 11, 12), which probably resulted from the episodic northward subduction events that have been partially terminated and relocated by obductions. However, the exact geometry of the Mesozoic trenches south of Chukotka and Arctic Alaska remains to be clarified. A southward-dipping subduction might have existed as well and would have been a more efficient mechanism to explain continental ribbon detachment (by slab pull) and subsequent collision with the Siberian Craton (in the same manner as the Paleotethys closure formed the Neotethys). This model is supported by volcanism south of the South Anyui Suture (on Anyui-Svyatoinos arc, see Zonenshain et al. 1990) and implies the existence of a set of trenches with reversed polarities, a configuration found today in the SW Pacific.

## Summary and Concluding Remarks

The High Arctic region is a collage of young and old oceanic basins, with blocks of continental slivers scattered among them. New data cannot completely uncover the complexity of this area, but observational evidence appears to be consistent with the following interpretations:

A. The Eurasian Basin is undoubtedly floored by oceanic crust. Based on potential field data, the margins seem to have experienced a short-lived break-up, but transitional-type crust (with serpentinite ridges, e.g., Minakov et al. 2012) or an older-than-C24 opening (Brozena et al. 2003) are possible interpretations for the Eurasian flank of the Lomonosov Ridge. Although a long-lived mega strike-slip boundary along the entire Nares Strait length is now discounted, a possible connection with the Baffin Bay/Labrador Sea is not excluded as suggested by large-scale plate kinematics.
B. Shortly before the opening of the Eurasia Basin, extension is predicted by large-scale plate tectonic models (e.g., Gaina et al. 2002), and this could have triggered continental splinters from the Amerasian side of the Lomonosov Ridge to be detached from the ridge and form small basins, floored by either extended continental crust or even oceanic crust (for example, Makarov and possibly part of the Podvodnikov Basin).
C. Pseudo-linear magnetic anomalies, a sinuous feature that can be interpreted from potential field data as an extinct ridge, and a thin crust predicted by the gravity anomaly inversion are consistent with an oceanic type crust in the Canada Basin. Although a definitive consensus toward the mechanism of this basin formation is not reached, a “rotational” model is partially supported. A “bipolar” opening due to southward and northward-directed subduction along the South Anyui and Angaucham trenches could provide a viable mechanism for detaching several continental blocks and to form the Amerasian Basin. Global tomography models image the slab graveyard below the South Anyui Suture and its surface expression, but more sophisticated methods are required to extract all the information from these data and models.
D. The timing and the extent of High Arctic Large Igneous Province (HALIP) is not well matched by paleopositions of the Iceland plume. However, part of the HALIP was formed at times when the Plume Generation Zone (Torsvik et al. 2006) was located beneath the North Atlantic and High Arctic regions.

Numerous questions regarding the configuration of High Arctic remain unanswered. Among them are the nature of the Alpha Ridge and timing and extent of volcanism and a proper understanding of the terrane amalgamation around the High Arctic region (NE Asia, North Pacific and NW America). In this study, we have used only the freely available data and published results but, in the near future, the access to the wealth of data collected in the Arctic in the last decade as well as new data collected for “Law of the Sea” exploration will unquestionably shed more light upon the unresolved issues.
